# The effects of weight loss on health-related quality of life in obese women with PCOS and controls

**DOI:** 10.1186/s12905-023-02682-9

**Published:** 2023-10-10

**Authors:** Farnaz Shishehgar, Fahimeh Ramezani Tehrani, Setareh Vahidi

**Affiliations:** 1 Present Address: Department of Midwifery, Faculty of Nursing and Midwifery, Tehran Medical Sciences, Islamic Azad University, Tehran, Iran; 2 Present Address: Reproductive Endocrinology Research Center, Research Institute for Endocrine Sciences, Shahid Beheshti University of Medical Sciences, Tehran, Iran; 3 Present Address: School of Medicine, Iran University of Medical Sciences, Tehran, Iran

**Keywords:** Weight loss, Dietary intervention, PCOS, Obesity, HRQOL

## Abstract

**Background:**

Polycystic ovary syndrome (PCOS) is a complicated endocrine disorder with widespread symptoms that reduce women’s quality of life. The adverse effect of associated obesity on this reduction is unclear, therefore the impact of weight loss on health-related quality of life (HRQOL) in obese women with PCOS is unknown. This study aimed to compare the impact of weight loss following a 24-week dietary intervention on HRQOL in obese women with and without PCOS.

**Methods:**

In a 24-week hypocaloric LGI (low glycemic index) diet intervention study, 286 women were recruited (140 PCOS, 146 controls) and 216 participants (PCOS = 105, non-PCOS = 111) completed the study. HRQOL was assessed using the SF-36 questionnaire (Short Form Health Survey). Physical activity was measured using the short form of the International Physical Activity Questionnaire (IPAQ). Anthropometric assessments, dietary intake, physical activity levels, and HRQOL scores, according to the Iranian version of SF-36, were compared at baseline and 24 weeks with intervention.

**Results:**

At the end of the intervention, there was no significant difference in the percentages of weight loss between the two groups (PCOS: 6.29 ± 3.32% vs. non-PCOS: 7 ± 3.62%, p = 0.1). At baseline, women with PCOS had lower mean scores in aspects of physical function (PF), general health perception (GH), role limitation due to emotional problem (RE), vitality (VT), mental health (MH), physical (PCS) and mental component summary scales (MCS), compared to non-PCOS (P < 0.01). At 24 weeks, the percentage of improvement in HRQOL in the non-PCOS group was higher compared to the PCOS group but this difference didn’t reach statistical significance except for PCS. In both groups, greater weight loss was associated with further improvement in the physical aspects of HRQOL and vitality. In the non-PCOS group, with trivial exception, greater weight loss was associated with greater improvement in the mental aspects of HRQOL.

**Conclusions:**

Both obese PCOS and non-PCOS women achieved nearly similar levels of improvement in HRQOL due to weight loss induced by a hypocaloric LGI diet.

**Trial registration:**

This study is registered in the Iranian Randomized Clinical Trials Registry (IRCT, code: IRCT2016092129909N1).

## Background

Polycystic ovary syndrome is a multisystem disorder in women of reproductive age that influences the metabolic, reproductive, and mental aspects of women’s lives. The characteristic features of PCOS encompassing hirsutism, oligomenorrhoea, and obesity impair quality of life [1].

Current evidence suggests obesity has a mutual association with PCOS and can deteriorate clinical, reproductive, metabolic, and psychological features of PCOS [2]. The incidence of PCOS in Iranian women based on criteria of the National Institute of Health (NIH), Rotterdam, and the Androgen Excess Society (AES) were 7.1%, 14.6%, and 11.7%, respectively [3]. Up to 60% of women with PCOS are either overweight or obese and obesity may by itself, diminish the HRQOL of women [4, 5]. HRQoL is a multidimensional concept that includes an individual’s own evaluation of physical, mental, social, and other aspects of health [6]. It is defined as an “individual’s perception of their own life in the context of their cultures and beliefs, and their personal goals and concerns” [7].

Despite the high prevalence of obesity in women with PCOS, few studies have investigated the effects of dietary weight loss intervention in HRQOL of PCOS women [8–13].

Obesity is one of the major health issues in this century. World Health Organization declared the prevalence of obesity has doubled since 1980 [14]. The prevalence of overweight and obesity in Iranian women was 35.2% and 30.4% respectively [15]. Prior investigations reported that obesity not only increases the risks associated with chronic diseases but also reduces HRQOL [16].

Current evidence regarding the impact of dietary weight loss interventions to change generic HRQOL among the general population is scarce and inconsistent as some trials show improvements while others do not. Inconsistencies in the relationship between weight reduction and improved HRQoL are ascribed to the following factors: the heterogeneity in weight loss interventions, various HRQoL measures, diverse types of interventions, various study populations, poor quality of reporting of HRQoL outcomes (as the primary outcome is weight loss rather than HRQoL) [17, 18]. In dietary interventions, the beneficial effect of weight loss on subscales of generic HRQL varied across the studies. Some studies have reported improvements in PCS [19] and MCS following dietary interventions [20], while others have found improvements in various subscales of HRQOL [21–24]. However, some have shown no significant improvement in the subscales of HRQOL [25].

Studies conducted on women with excess weight demonstrated that regardless of PCOS status, 5–10% of weight loss has similar positive effect on the anthropometric and metabolic characteristics of women with and without PCOS [26]; however, a limited number of studies compared its beneficiary effect in terms of HRQL between women with PCOS and healthy controls. To the best of our knowledge, this is the first study among Iranian women that compared the impact of dietary weight loss intervention on HRQOL between women with and without PCOS.

Moreover, most studies about the influence of weight loss on HRQOL have focused on combined obesity treatments including anti-obesity medications combined with dietary intervention or bariatric surgery, and conducted in Western countries. Although the results of dietary interventions suggest that the percentage of weight loss achieved by both the PCOS women and non-PCOS women are the same, it is not clear if obese PCOS women experience the same improved HRQOL after weight loss. Therefore, the aim of this study is to compare the effect of weight loss following the hypocaloric low glycemic index (LGI) diet on HRQOL in women with and without PCOS.

## Methods

### Study design

In this interventional study, participants, PCOS, and controls, who fulfilled the study inclusion/exclusion criteria received a 24-week reduced energy low glycemic diet.

Study participants were assessed at baseline and six months after diet. Written informed consent was obtained from all participants before the commencement of the study and ethical approval was acquired from the ethics committee of the Research Institute of Endocrine Sciences (approval no: IR.SBMU.ENDOCRINE.REC.1395.276).

This study is registered in the Iranian Randomized Clinical Trials Registry (Clinical trial registration number: IRCT2016092129909N1). The date of the first registration was 08/12/2016.

### Participants and setting

This study was carried out at the Reproductive Endocrinology Research Center, Research Institute for Endocrine Sciences, Shahid Beheshti University of Medical Sciences (SBUMS), from 4/1/ 2019 to 16/2/2020. Two hundred eighty-six overweight/obese women, aged 18 to 40 years, including 140 PCOS and 146 age and BMI-matched healthy eumenorrheic, non-hirsute controls, were recruited. In this study, 37.1% (n = 52) of the PCOS group had phenotype A, 26.4% (n = 37) had phenotype B, 13.6% (n = 19) had phenotype C, and 22.9% (n = 32) had phenotype D.

Four health centers were randomly selected from the centers affiliated with SBUMS. The announcement of the weight loss project, the conditions for its implementation, and the information of the principal investigator were posted on the notice boards of the selected clinics and the Reproductive Endocrinology Research Center. The dietary intervention and follow-up methods were explained to the volunteers, and after obtaining informed consent, they participated in the study. Age and BMI of the non-PCOS group were matched to those of the PCOS cases. The PCOS group was divided into three age groups (< 25, 25–30, and > 30 years) and two BMI groups (26–30 and > 30 kg/m^2^), resulting in six age and BMI subgroups for PCOS cases. Finally, a total of 286 women, including 140 with PCOS and 146 BMI and age-matched controls without PCOS, agreed to participate in the study.

Participants with a confirmed diagnosis of PCOS (cases) were selected from the Reproductive Endocrinology Research Center of SBUMS and controls were recruited from among women who attended health centers for their routine gynecological exams. PCOS was determined based on the Rotterdam criteria as defined by the presence of two or more of the following: (1) Oligo- and/or anovulation, (2) clinical or biochemical hyperandrogenism (3) Polycystic ovaries (PCO) [27].

The inclusion criteria were age (between 18 and 40 years), BMI between 26 − 38 kg/m^2^, ability to read and write, and weight stability for 3 months before the intervention.

Study exclusion criteria included pregnancy, breastfeeding, using lipid-lowering agents, insulin-sensitizing drugs, contraceptive drugs, special diets or exercise for weight loss, and antipsychotic or hormonal medications during the previous 6 months. Participants were also excluded if they had histories of mental disease and malignancy.

### Dietary intervention

A hypocaloric low glycemic index (LGI) diet was computed for a BMI of 22 kg/m^2^ and a deficit of 500 kcal that induced nearly 0.5 kg weight loss per week. The targeted nutritional plan included 50% of energy from carbohydrates (CHO) with low GI, 30% of energy from fat, and 20% of energy from protein. A list of high Glycemic Index foods was prepared and the participants were prohibited from eating any high GI items. All participants were trained to record their daily dietary intake by a qualified nutritionist. Each participant received a personalized food menu based on eating habits and energy requirements. Once every two weeks, counseling visits were provided to participants to teach them how to record daily food intake. Adherence to the diet was defined by the percentage of energy supplied from carbohydrates, protein, and fat. Dietary intake was computed from a 3-day dietary record biweekly. The Nutritionist IV software was used to calculate the energy and nutrient intake of each food item.

### Anthropometric measurements

All anthropometric assessments were taken in the morning after overnight fasting. Weight was measured with minimal clothing, and bare feet using a digital scale (Seca, Hamburg, Germany) with an accuracy of 100 g. Height was measured using a Stadiometer (SECA 213) in a standing position with bare feet, placing the hands next to the body, looking straight ahead, shoulders relaxed, and in a position where the shoulder blades, buttocks, and heels are in contact with the Stadiometer with an accuracy of one millimeter. Waist circumference was measured with a non-stretchable tape without imposing any pressure on the body and at the narrowest part of the waist between the last rib and the iliac crest (to the nearest 0.1 cm) at the end of a normal exhalation. Hip circumference was measured (to the nearest 0.1 cm) in a standing position at the widest part of the buttocks using non-stretchable tape. Body mass index (BMI) was estimated by dividing weight in kilograms by the square of the height in meters.

### Questionnaires

Socio-demographic information was assessed with a questionnaire that included 5 questions about age, marital status, education level, employment, and residence.

The Short Form Health Survey (SF-36) is a widely known tool to measure health concepts [28]. In this study, The Iranian version of the SF-36 was utilized [29]. It has been validated for use in assessing health-related quality of life in Iran [29]. It consists of eight domains; physical functioning (PF), role limitation due to physical problem (RP), bodily pain (BP), general health perception (GH), vitality (VT), social functioning (SF), role limitation due to the emotional problem (RE) and mental health (MH). These eight dimensions can be summarized in two summary scales: the physical (PCS) and mental component summary scales (MCS), dimensions which are scored from 0 (the minimum possible health status assessed by the questionnaire) to 100 (the maximum possible health status). Validity and reliability of the Short Form 36-item questionnaire for Iranian adults have been approved, with convergent validity ranging from 0.58 to 0.95 and Cronbach’s alpha level ranging from 0.77 to 0.90 (alpha = 0.65) [29].

All participants were asked to maintain their physical activity. Physical activity was measured by the International Physical Activity Questionnaire (IPAQ). It is a self-reported measure of physical activity that is appropriate for evaluating population levels of physical activity [30]. The validity and reliability of IPAQ have been confirmed [30].

The participants were requested to complete the short form of the International Physical Activity Questionnaire (IPAQ) every 4 weeks. This questionnaire consists of 7 items about vigorous, moderate physical activity, and walking time during the previous week [30]. The IPAQ guideline was used to assess physical activity which scores Met level of 8 for vigorous, 4 for moderate intensity, and 3.3 for walking. Anthropometric assessments, dietary intake, physical activity levels, and HRQOL scores were compared at baseline and 24 weeks with intervention.

### Statistical analysis

To calculate the sample size, we assumed a difference of 2.01 (7.67) and − 1.3 (8.55) for the PCOS and control group, respectively [12]. A total number of 202 participants (101 in each group) was adequate to identify a difference in PCS in cases compared to controls, with a power of 80%, α = 0.05, and a dropout rate of 20%.

Participants were classified based on the percentage of weight loss throughout the time of intervention: weight loss of 0-4.99% (category 1), weight loss of 5-9.99% (category 2), and weight loss of > 10% (category 3). Categorical variables are presented as percentages and the Chi-square test was used to compare among study groups. One sample Kolmogorov-Smirnoff test and the normal plot were utilized to test the normality of continuous variables. Baseline characteristics of study groups and the percentage change in dimensions of HRQOL from baseline to six months were illustrated as mean ± standard deviation.

The independent T-test was used to compare the differences between cases and controls and Mann–Whitney U-test was applied for non-normal distributed data. To define within-group differences, paired T-test was used or Wilcoxon signed-rank test for data with skewed distributions. Kruskal Wallis and Mann–Whitney U-test were applied to compare HRQOL changes classified by percent of weight change. In post hoc analysis, Bonferroni correction was performed to adjust the p-value, when we used three separate comparisons. Thus, results were regarded as significant at a p-value of < 0.01. We applied statistical analysis using IBM SPSS Statistics (Version 20) [31].

## Results

### Participant characteristics

Of 286 obese women selected for the study, 23 were withdrawn before the initiation of the study, thus 263 participants including 128 with PCOS and 135 without PCOS received the diet. Of these participants, 105 women with PCOS and 111 without PCOS completed a 24-week hypocaloric LGI diet. The mean BMI and age in the PCOS group were (31.04 ± 3.62 kg/m^2^, 28.5 ± 4.3 years) and in the controls (30.7 ± 3.45 kg/m^2^, 29.5 ± 5.2 years). At baseline, there were no statistically significant differences in the socio-demographic and anthropometric characteristics of cases and controls except for waist circumference (P < 0.001), and waist-to-hip ratio (P < 0.001) (Table 1).

**Table 1:** Characteristics of study participants

Characteristics	PCOS (n = 105)	Controls (n = 111)	p-value
Age (years)	28.50 ± 4.30	29.50 ± 5.20	0.100
Education, n (%)			
High school Diploma and higher	29 (27.6%) 76 (72.4%)	27 (24.3%) 84 (75.5%)	0.300
Employment			
Employed Unemployed	39(37.1%) 66 (62.9%)	46 (41.4%) 65 (58.6%)	0.500
Marital status			
Single Married	34 (32.4%) 71 (67.6%)	25 (22.5%) 86 (77.5%)	0.070
BMI (kg/m^2^)	31.04 ± 3.62	30.70 ± 3.45	0.400
BMI category, n (%)			
Overweight	47 (44.8%)	50 (45%)	
Obese	58 (55.2%)	61 (55%)	0.200
Waist circumference (cm)	100 ± 10.42	93.42 ± 13.70	0.001
Waist-to-hip ratio (cm)	0.89 ± 0.04	0.79 ± 0.07	0.001
Weight loss, n (%)			
0-4.99%	33 (31.4%)	30 (27%)	
5-9.9% 10%.	62 (59%) 10 (9.5%)	55 (49.5%) 26 (23.4%)	0.020
Weight loss (kg)	− 5.10 ± 2.70	-5.79 ± 3.36	0.090
Weight loss% (kg)	- 6.29 ± 3.32	− 7 ± 3.62	0.100

Data are presented as mean (SD) or number (percentage)

Independent t-test or Mann–Whitney U test was used for continuous variables and Pearson’s χ2 test for categorical ones.

Kg, Kilogram, cm, Centimeter

Significant difference (P < 0.05)

Because there was no significant difference in socio-demographic characteristics between the two groups, we didn’t adjust SF-36 scale scores for the impact of socio-demographic characteristics.

The attrition rate was similar in both groups (PCOS = 23 (17.97%) vs. non-PCOS = 24, 17.8%) and there were no significant differences in baseline data between completers and those who dropped out. At baseline, dietary intakes and physical activity levels of PCOS women and controls were the same (Table 2).

**Table 2:** Dietary intakes and physical activities of women with polycystic ovary syndrome compared to controls following a 24-week hypocaloric low glycemic index diet

Variables	Baseline	p-value (Baseline)	24 weeks	Δ 24–0 weeks	Within group p-value	Between group p-value
Energy (Kcal)						
PCOS (n = 105) Controls (n = 111)	2318.3 ± 374.2 2279 ± 280	0.500	1442.5 ± 101.6 1453.4 ± 108	-875.8 ± 394.3 -825.5 ± 295.4	< 0.001	0.400
Carbohydrate (%)						
PCOS (n = 105) Controls (n = 111)	54.8 ± 12.4 56.3 ± 4.7	0.400	49.9 ± 6 50.6 ± 5	-4.8 ± 13.8 -5.6 ± 7	< 0.001	0.400
Carbohydrate (gram/day)						
PCOS (n = 105) Controls (n = 111)	310.4 ± 56.8 320.9 ± 48.6	0.100	180.6 ± 28.9 184.6 ± 26.7	-129.7 ± 64 -136.2 ± 56.8	< 0.001	0.500
Protein (%)						
PCOS (n = 105) Controls (n = 111)	10.1 ± 2 10.2 ± 1.5	0.500	19.9 ± 4.1 20 ± 4.3	9.7 ± 4.1 9.8 ± 4.4	< 0.001	0.700
Protein(gram/day)						
PCOS (n = 105) Controls(n = 111)	57.9 ± 11.4 58.3 ± 9.5	0.700	71.6 ± 13.9 72.8 ± 15.4	13.7 ± 17.3 14.4 ± 17.3	< 0.001	0.500
Fat (%)						
PCOS (n = 105) Controls (n = 111)	35 ± 13.4 33.3 ± 4.9	0.200	30.1 ± 3.2 29.2 ± 3.4	-4.9 ± 13.7 -4.1 ± 5.7	< 0.001	0.400
Fat (gram/day)						
PCOS (n = 105) Controls (n = 111)	93.8 ± 45.8 84.6 ± 17.5	0.060	48.1 ± 4.4 47 ± 5	-45.7 ± 40.9 -37.6 ± 17.5	< 0.001	0.400
GI						
PCOS (n = 105) Controls (n = 111)	59.9 ± 5.7 59.2 ± 7.1	0.100	42.6 ± 11.3 44.2 ± 9.4	-17.2 ± 12.5 -14.9 ± 11	< 0.001	0.100
GL						
PCOS (n = 105) Controls(n = 111)	147.9 ± 15.6 153.7 ± 33.1	0.200	66 ± 23.6 69.7 ± 20.4	-81.9 ± 28.8 -83.9 ± 37.3	< 0.001	0.600
Physical activity (Met- minute_week)						
PCOS (n = 105) Controls (n = 111)	456.4 ± 112.6 438.9 ± 115	0.300	441.3 ± 123.6 443.7 ± 105.3	-15 ± 159.4 4.7 ± 141	0.300	0.200

Data are presented as mean ± SD and number (%). The independent T-test or Mann–Whitney U-test was used to compare the differences between cases and controls. Paired T-test or Wilcoxon signed-rank test was used to define within-group differences

PCOS Polycystic ovary syndrome, Kcal Kilocalories, GI Glycemic index, GL Glycemic load

Significant difference (P < 0.05)

The mean scores in diverse aspects of HRQOL of women with and without PCOS were illustrated in Table 3. Women with PCOS had significantly lower mean scores in dimensions of PF, GH, RE, VT, MH, PCS, and MCS than those of non-PCOS. In PCOS women the lowest scores were reported on the mental health, vitality, and role emotional domains of the SF-36 (Table 3).

**Table 3:** Comparisons of means and standard deviations for sub-scores of health-related quality of life dimensions between women with polycystic ovary syndrome and controls

Variables	Baseline	P value (Baseline)	24 weeks	Δ 24–0 weeks Percentage change mean (SD)	Within group p-value	Between group p-value
PF						
PCOS (n = 105) Controls (n = 111)	62.80(24.50) 71.70 (20.40)	0.010	69.10(18.90) 83.10 (13.50)	18.98(33.40) 22.92(30.60)	0.001 0.001	0.300
RP						
PCOS (n = 105) Controls (n = 111)	66.40 (24.70) 71.50 (13.90)	0.900	69.10 (20.90) 79.20 (10.70)	11.49(26.90) 13.70(18.40)	0.001 0.001	0.400
BP						
PCOS (n = 105)	67.79 (27.60)	0.700	69.28(23.90)	7.59(22.40)	0.400	
Controls (n = 111)	71.79 (17.10)		75.94(13.60)	8.59(14.80)	0.001	0.600
GH						
PCOS (n = 105) Controls (n = 111)	66.06 (18) 77.86(14.90)	0.001	71.78(14.40) 85.76(8.20)	12.45(20.60) 13.68(21.10)	0.001 0.001	0.600
RE						
PCOS (n = 105) Controls(n = 111)	54.14 (22.50) 68.66 (29.70)	0.001	53.54(21.50) 68 (27.60)	1.39(17.30) 1.75(13)	0.300 0.300	0.600
VT						
PCOS (n = 105) Controls (n = 111)	48.72 (22.50) 69.92 (15.20)	0.001	51.25(21) 76.99(12.10)	10.63(19.20) 12.23(13.60)	0.001 0.001	0.400
MH						
PCOS(n = 105) Controls (n = 111)	46.33(22.60) 72.96 (15.70)	0.001	46.59(20.80) 71.57(11.50)	4 (17.10) 0.33(14.20)	0.400 0.100	0.080
SF						
PCOS (n = 105)	69.59 (27.80)	0.200	68.97(24.80)	2.10(25.60)	0.400	
Controls (n = 111)	75.56 (17.70)		72.71(14.10)	− .37(22.40)	0.070	0.400
PCS						
PCOS (n = 105) Controls (n = 111)	65.80 (14.70) 73.24(12.30)	0.001	69.83(12.30) 81(6.90)	7.79(12.20) 12.63(13.40)	0.001 0.001	0.006
MCS						
PCOS (n = 105) Controls (n = 111)	54.70 (17) 71.77 (13.70)	0.001	55 (15.50) 72.33(11.30)	2.23(9.40) 2.06(10.10)	0.300 0.200	0.300

Data are presented as mean ± SD and number (%)

Significant difference (P < 0.05)

### Dietary intake and physical activity

At the end of the intervention, we defined a significant decline in the consumption of energy (PCOS =-875.8 ± 394.3, non-PCOS = -825.5 ± 295.4 kcal/day, p = 0.4), GI (PCOS = − 17.2 ± 12. 5, non-PCOS = − 14. 9 ± 11, p = 0.1) and GL (PCOS = − 81.9 ± 28.8, non-PCOS = − 83.9 ± 37.3, p = 0.6) which were significant within each group, but did not differ significantly between groups. After the intervention, physical activity levels did not differ significantly in either of the two groups (Table 2).

### Weight loss

At six months, both groups achieved similar weight reduction. The mean weight reduction is 5.1 ± 2.7 kg for PCOS women and 5.79 ± 3.36 kg for non-PCOS women. In both groups, significant BMI reductions were reported, compared to baseline, in the PCOS group (31.04 ± 3.62 kg/m^2^ vs. 29.09 ± 3.58 kg/m^2^, p < 0.001) and in controls (30.07 ± 3.45 kg/m2 vs. 28.4 ± 2.8 kg/m^2^, p < 0.001) (Table 4). The percentage of reduction in BMI didn’t differ between the two groups (-%6.29 ± 3.32 for the PCOS group and - % 7 ± 3.62 for the non-PCOS group, P = 0.1).

**Table 4:** The effect of a 24-week dietary intervention on anthropometric variables in women with polycystic ovary syndrome compared to controls

Variables	Baseline	P value (Baseline)	24 weeks	Δ 24–0 weeks	Within group p-value	Between group p-value
Weight (kg)						
PCOS (n = 105) Controls (n = 111)	81.10 ± 11.70 79.80 ± 11.70	0.400	76 ± 11.40 74 ± 9.20	-5.09 ± 2.70 -5.79 ± 3.36	0.001 0.001	0.090
Waist circumference (cm)						
PCOS (n = 105) Controls (n = 111)	100 ± 10.40 93.40 ± 13.70	0.001	92.40 ± 10.80 85.60 ± 10.60	-7.65 ± 5.50 -7.79 ± 6.54	0.001 0.001	0.800
Waist-to-hip ratio (cm)						
PCOS (n = 105) Controls (n = 111)	0.89 ± 0.04 0.79 ± 0.07	0.001	0.87 ± 0.04 0.78 ± 0.06	-0.02 ± 0.04 -0.009 ± 0.04	0.001 0.030	0.001
BMI (kg/m^2^)						
PCOS (n = 105) Controls (n = 111)	31.04 ± 3.62 30.07 ± 3.45	0.400	29.09 ± 3.58 28.40 ± 2.80	-1.95 ± 1 -2.21 ± 1.25	0.001 0.001	0.090

Data are presented as mean ± SD

Significant difference (p < 0.05)

The classification of weight change of both groups is portrayed in Table 1. Most participants achieved weight reductions of 5–9.9% (59% for the PCOS group and 49.5% for the non-PCOS group). Out of 105 women with PCOS, 68.5% (n = 72) lost more than 5% of their initial weight and 31.4% (n = 33) lost less than 5% of their baseline weight. Out of 111 healthy women, 72.9% (n = 81) lost more than 5% of their initial weight and 27% (n = 30) lost less than 5% of their baseline weight.

### HRQOL changes

In the PCOS group, we noticed a significant enhancement in mean scores of PF, GH, RP, VT, and PCS at six months of intervention. In controls, the HRQOL improved significantly in all dimensions of physical HRQOL, PCS, and vitality. In both groups, the greatest improvement was in the physical function of HRQOL; however, the percentage change comparison between groups was not statistically significant. There was no statistically significant difference between the percentage change of HRQOL in the PCOS group compared to the non-PCOS group except for PCS (7.79 ± 12.21 vs. 12.63 ± 13.42, p = 0.006). With trivial exceptions, mean percentage changes in HRQOL were lower in women with PCOS compared to non-PCOS women, however, the percentage change comparison between groups was not statistically significant. In both groups, there was no significant improvement in the mental component score of HRQOL (Table 3).

The mean percentage change in HRQOL regarding weight loss classification is illustrated in Table 5.

**Table 5:** Comparison of the change of HRQOL by weight loss classification between cases and controls

Variable	PCOS(n = 105) Baseline mean (SD)	PCOS(n = 105) 24 weeks mean (SD)	Controls (n = 111) Baseline mean (SD)	Controls (n = 111) 24 weeks mean (SD)	Percentage change of HRQOL mean (SD) in cases at 24 weeks	Percentage change of HRQOL mean (SD) in controls at 24 weeks	Between groups p-value
PF							
0.1–4.9% loss 5%-9.9% loss 10–15% loss	64.62 (24) 61.15 (24.70) 67.50 (26.40)	63.20(19.30) 70.10(18.20) 82(14.90)	81(13.20) 74(21.10) 56.15(17.40)	85.50(14.10) 87.80(10.50) 70.60(11.20)	3.73(30.60) ^a, b, c^ 24.99(32.70) 32.09(32.30)	5.80(8.70) ^a, b, c^ 26.90(32.60) 34.23(34.80)	0.10 0.90 0.90
RP							
0.1–4.9% loss 5%-9.9% loss 10–15% loss	70.60(24.90) 63.33(25.80) 72.50(11.80)	69(20.70) 66.50(21.10) 85.50(12.10)	75.16(9) 73(14) 64.23(16.20)	77.40(7.30) 82.80(10.70) 73.80(11.40)	4.43 (27.70) ^a, b, c^ 14.10(27.90) 18.50 (8)	4.10(12.30) ^a, b, c^ 16.10(17.60) 19.58(22)	0.90 0.60 0.80
BP							
0.1–4.9% loss 5%-9.9% loss 10–15% loss	70.48(28.80) 65.95(29.20) 70.25(7.60)	68.30(25.50) 67.90(24.50) 81(8)	81.25(15.69) 66.44(18.60) 72.21(9.20)	79.60(13.80) 71.10(14.20) 81.70(7.20)	0.47(15.50) ^a, c^ 10 (25.90) 15.70 (9)	-1(8.20) ^a, b, c^ 11.26(16.80) 14.10(10.70)	0.50 0.70 0.60
GH							
0.1–4.9% loss 5%-9.9% loss 10–15% loss	65.93(16.40) 67.91(18.30) 55(18.40)	63.70(14.30) 76.80(11.50) 66.50(18.40)	84.86(15.20) 76.96(14.50) 71.69(12.70)	80.60(9.90) 88.10(7) 86.50(5.40)	-1.64(13.40) ^a, b, c^ 18.34 (21.30) 22.50 (9.90)	-2.74(15.70) ^a, b, c^ 17.77(19.10) 23.96(20.30)	0.70 0.80 0.70
RE							
0.1–4.9% loss 5%-9.9% loss 10–15% loss	54.27(26.10) 53.47(20.5) 57.90(24.3)	52.80(24.40) 53.70(19.80) 54.20(23.30)	68.99(28.50) 68.36(29.70) 68.90(32)	67.60(25.60) 68.90(27.70) 66.70(30.40)	-0.35 (12.10) 3.24 (19.60) -4.28 (15.90)	-0.33(12.50) 3.60(12.60) -0.69(14)	0.80 0.50 0.50
VT							
0.1–4.9% loss 5%-9.9% loss 10–15% loss	49(21.60) 47.25(22.50) 56.83(26.30)	49.10(19.90) 50.50(20.90) 62.80(24.10)	77.16(17.60) 69(14.10) 63.30 (10.60)	78.80(17.10) 76.50(10) 75.76(9.50)	3.43(12.20) ^a, b, c^ 12.46(17.20) 23(36.70)	2.67(4.70) ^a, b, c^ 13.31(14.90) ^b, c^ 20.94(10.70)	0.06 0.70 0.70
MH							
0.1–4.9% loss 5%-9.9% loss 10–15% loss	40.93(22.20) 46 (22.40) 65.60(15.30)	39.20(17.50) 46(19.80) 74.50(14.40)	80.36(11.10) 70.58(17.30) 69.46(14.50)	77.40(9.40) 68.50(12.30) 74.60(10.90)	1(16.90) 3.86(17.20) 15(14.40)	-7(6.30) ^a, c^ -0.04(15.60) ^b, c^ 9.61(12.60)	0.02 0.20 0.20
SF							
0.1–4.9% loss 5%-9.9% loss 10–15% loss	73.71(19.60) 66.73(31.30) 73.75(27.90)	73.10(18) 65.70(27.90) 75(22.30)	87.41(12.70) 71.36(18) 70.76(16.20)	80.50(11.50) 66.10(13.50) 77.50(11.20)	0.44(11.80) -0.12(14.40) 20.46 (71.40)	-6.79(13.50) ^a, c^ -4.20(19.30) ^b, c^ 15.15(29.20)	0.02 0.20 0.70
PCS							
0.1–4.9% loss 5%-9.9% loss 10–15% loss	67.91(10.90) 64.59(14.40) 66.31(13)	66.10(14) 70.30(10.90) 78.70(9.90)	80.57(10.20) 72.63(12.50) 66 (9.30)	80.80(7.50) 82.40(7.10) 78.20(4.80)	-1.90 (6.60) ^a, b, c^ 10.93(11.20) ^b, c^ 20.39(11)	0.95(6.40) ^a, b, c^ 15.58(12.80) 19.87(12.20)	0.10 0.08 0.80
MCS							
0.1–4.9% loss 5%-9.9% loss 10–15% loss	54.48(15.90) 53.39(18) 63.52(11.80)	53.60(14.10) 54 (16.30) 66.60(10.20)	78.48(10.90) 69.84(14.20) 68.11(13.10)	77.28(10.60) 72.21(11.10) 75.50(10)	− .056 (7.20) 3.14 (10.60) 5.82(7.37)	-4 (4.80) ^a, b, c^ 1.70(9.40) ^b, c^ 9.70(11.10)	0.02 0.40 0.30

Kruskal Wallis and Mann–Whitney U-test were applied to compare HRQOL changes classified by percent of weight change. Different letters showed significant differences in the percentage change of HRQOL in the same group (p < 0.01)

The Mann–Whitney U-test was used to compare the differences between cases and controls

Significant difference (p < 0.01)

In both groups, there was an increasing tendency in HRQOL across the weight loss classification, greater weight reduction was associated with a rising improvement in the physical aspects of HRQOL and vitality. In the non-PCOS group, with trivial exception, greater weight loss was associated with greater improvement in the mental aspects of HRQOL.

## Discussion

This study revealed that despite observing lower mean scores of various aspects of HRQL in women with PCOS at baseline, both PCOS and non-PCOS groups experienced a similar improvement in HRQOL after receiving a 24-week hypocaloric low glycemic index diet except for PCS which improvement was significantly higher among non-PCOS participants. In both groups, greater weight loss was associated with more improvement in HRQOL.

It is evident that BMI wouldn’t be the isolated definitive factor to deteriorate the HQOL in PCOS women, regarding those studies comparing HRQOL between women with PCOS and controls, showed various aspects of HRQOL were worsened in PCOS women [32, 33]. Our findings are contrary to those studies reporting no independent association between PCOS and reduced general HRQOL [34].

We noted that after six months of dietary intervention, both groups had a noticeable improvement in HRQOL. Similar to our findings, a recent study demonstrated weight loss had similar positive changes in the HRQOL of women with and without PCOS [35].

In both groups, the percentage change in HRQOL scores was related to the amount of weight loss. A higher percentage of improvement in physical aspects of HRQOL was reported in participants who achieved greater weight reduction (> 5%). In the non-PCOS group, with greater weight loss, an increasing tendency to improve the mental aspects of HRQOL was noted.

Our results are not in agreement with those of results that reported a lack of relationship between enhancements of HRQOL with weight loss [36], a controversy due to the amount of participants’ weight loss; in the aforementioned study, 62% of the study population achieved a weight loss of < 5%. In our investigation, 71% of participants achieved a weight loss equivalent to 5 to 10% of their initial weight.

Unlike our findings, a randomized control trial demonstrated that weight reduction had no significant positive effect on subscales of HRQOL, a difference that may be related to participants’ age in the latter study, the mean age of participants was 60 years old. Furthermore, at baseline, women participating in that trial had high scores of HRQOL so there was little room for enhancement [25].

In both groups, a remarkable improvement in HRQOL was reported with regard to aspects of PF, RP, GH, VT, and PCS in participants who had lost ≥ 5% of their initial weight. A population-based study revealed at least 5–10% weight loss was related to improved HRQOL [37].

We demonstrated that the greatest improvement occurred in the physical function of HRQOL.

This result may be related to the negative effect of obesity on mobility and musculoskeletal disorders [38]. Previous studies have shown that rising BMI leads to pain and a decrease in physical function [39].

The results of our study are consistent with a Meta-analysis that shows physical and mental aspects of HRQOL were adversely influenced by obesity however this influence is greater on the physical dimensions of HRQOL [40]. The results of other interventional studies documented such associations and reported that weight reduction had a greater impact on the physical than mental dimensions of HRQOL [41, 42].

A review article including 20 studies revealed significant improvement in physical aspects rather than mental health components after weight reduction, however, they mainly included research on HRQOL due to surgical interventions rather than dietary interventions [18]. Despite this review, we found that weight loss after dietary intervention had a positive effect on the physical aspects of HRQOL and vitality. Among the mental aspects of HRQOL, the significant improvement due to weight loss occurred in the domain of vitality in both groups, findings are consistent with studies that reported the vitality aspect of HRQOL is sensitive to weight reduction [21].

Our study has several strengths. We used the SF-36 questionnaire that was previously validated for Iranian women. We matched our participants for BMI and age, factors influencing the HRQOL. In most previous studies, weight loss was the outcome of dietary intervention, and sample size estimation was based on weight reduction whereas the outcome of the present intervention was HRQOL and the sample size calculation was based on the change in HRQOL scores. Therefore, compared to those trials, this study had greater power to detect enhancement in HRQOL. This study has some limitations; the participants were not blinded to the endpoint of the research so there is a risk of over-reporting of HRQOL measures. We have not considered some variables such as socio-demographic factors, self-esteem, depression, and weight loss expectations that may impact the alteration in HRQOL.

## Conclusions

Our findings have notable contributions to the scant evidence about comparing the impact of weight loss on HRQOL between obese PCOS women and obese non-PCOS controls.

Our findings showed similar improvement of HRQOL except for the physical component summary scale in obese women with PCOS compared to non-PCOS. In both groups, participants who achieved greater weight reduction had greater enhancement in HRQOL. In both groups, participants who lost at least 5%-10 of initial weight achieved noticeable advancement in the physical dimension of HRQOL.

Knowing that even modest weight loss following dietary intervention can lead to improving quality of life may cause to maintain motivation in obese women to follow weight loss programs.

## Data Availability

The datasets used and/or analyzed during the current study are available from the corresponding author on reasonable request.

## List of abbreviations

BMI: Body mass index
GI: Glycemic index
GL: Glycemic load
LGI: Low glycemic index
IPAQ: International Physical Activity Questionnaire
PF: physical functioning
RP: role limitation due to the physical problem
BP: bodily pain
GH: general health perception
VT: vitality
SF: social functioning
RE: role limitation due to the emotional problem
MH: mental health
PCS: the physical component summary scales
MCS: the mental component summary scales
PCOS: Polycystic ovary syndrome
